# Structural basis for E3 ubiquitin ligase UHRF1 binding to nucleosome core particle and histone H3 ubiquitination

**DOI:** 10.1016/j.jbc.2025.110894

**Published:** 2025-11-04

**Authors:** Reia Shikimachi, Shun Matsuzawa, Hiroki Onoda, Tsuyoshi Konuma, Atsushi Yamagata, Mikako Shirouzu, Kosuke Yamaguchi, Kyohei Arita

**Affiliations:** 1 Structural Biology Laboratory, Graduate School of Medical Life Science, Yokohama City University, Yokohama, Kanagawa, Japan; 2 Synchrotron Radiation Research Center, Nagoya University, Nagoya, Aichi, Japan; 3 Structural Epigenetics Laboratory, Graduate School of Medical Life Science, Yokohama City University, Yokohama, Kanagawa, Japan; 4 Laboratory for Protein Functional and Structural Biology, RIKEN Center for Biosystems Dynamics Research, Yokohama, Kanagawa, Japan; 5 Department of Chromosome Science, National Institute of Genetics, Research Organization of Information and Systems (ROIS), Mishima, Japan

**Keywords:** DNA methylation, cryo-EM, structural biology, nucleosome reconstruction, UHRF1, ubiquitination

## Abstract

The maintenance of DNA methylation in differentiated cells is regulated by a ubiquitin signal generated by UHRF1 (ubiquitin-like with plant homeodomain and RING finger domain 1), which plays a pivotal role in recruitment of DNA methyltransferase DNMT1 to hemimethylated CpG sites. UHRF1 catalyzes multiple monoubiquitinations on histone H3 within nucleosomes. However, the structural mechanisms underlying the binding of UHRF1 to nucleosomes and the subsequent formation of the ubiquitin signal remain incompletely understood. Here, we report cryo-EM structures of UHRF1 bound to nucleosome core particle (NCP) harboring H3K9me3 and a single hemimethylated CpG site. The structures of the UHRF1–NCP complexes reveal an unanticipated interaction between the UHRF1 tandem Tudor domain and the acidic patch of the NCP. This interaction enhances histone H3 ubiquitination and stabilizes UHRF1 binding to the NCP in a manner that is dependent on the position of the hemimethylated CpG site. These findings provide mechanistic insights into the binding of UHRF1 to the NCP and the multiple monoubiquitination of histone H3 within the NCP.

DNA methylation is one of the major epigenetic marks that predominantly occurs at the fifth carbon of cytosine residues within symmetric CpG dinucleotides, with approximately 70% to 80% of CpG sites in the mammalian genome being methylated (1, 2). DNA methylation is crucial for the regulation of gene expression, transposable element silencing, genomic imprinting, and X-chromosome inactivation, thereby contributing to cell fate and genomic stability (3, 4). Importantly, hemimethylated CpG sites, where only the template strand is methylated, are transiently produced after each round of DNA replication. To maintain their identity, cells faithfully inherit their DNA methylation patterns to daughter strands (5). DNA methylation maintenance involves two distinct processes: replication-coupled and replication-uncoupled maintenances (6, 7, 8). In both, E3 ubiquitin ligase UHRF1 (ubiquitin-like with plant homeodomain [PHD] and RING finger domain 1) plays a pivotal role in establishing a ubiquitin signal that recruits the maintenance DNA methyltransferase DNMT1 to hemimethylation CpG sites (9, 10). UHRF1 specifically recognizes hemimethylated CpG sites and ubiquitinates a replication factor PAF15 and histone H3 at early and late replication timings, respectively (6, 7, 11, 12, 13, 14). DNMT1 binds to ubiquitinated PAF15 and histone H3 *via* the replication foci targeting sequence domain (6, 14). This interaction releases the autoinhibitory structure of DNMT1 (15, 16, 17), resulting in the methylation of the target cytosine in the CpG sequence on the nascent strand. Aberrant DNA methylation patterns caused by dysregulation of DNA methylation maintenance are hallmarks of various cancer cells. These changes are often characterized by inappropriate silencing of tumor suppressor genes and increased genomic instability (18). Accordingly, both UHRF1 and DNMT1 have emerged as attractive therapeutic targets for epigenetic drug development in oncology (19, 20, 21).

UHRF1 has five functional domains—ubiquitin-like (UBL), tandem Tudor domain (TTD), PHD finger, SET and RING-associated (SRA) domain, and RING domain—connected by four intrinsically disordered linkers (Fig. 1*A*). The SRA domain is responsible for recruiting UHRF1 to DNA methylation sites by specifically binding to hemimethylated DNA (22, 23, 24). The TTD is a binding platform for various factors including dimethylated or trimethylated K9 in histone H3, methylated DNA ligase 1, and internal UHRF1 linkers (25, 26, 27, 28, 29, 30). The PHD finger recognizes the N-terminal sequences of histone H3 (^1^ARTK^4^) and PAF15 (^1^VRTK^4^), which are essential for their ubiquitination (6, 27, 31). The maternal factor DPPA3 antagonizes chromatin binding by targeting the PHD finger (32, 33, 34, 35). The UBL and RING domains are required for the ubiquitination activity of UHRF1 (36, 37). Crosstalk between DNA methylation and H3K9me3 has been shown to play a critical role in stable gene silencing, especially in late-replicating and pericentromeric heterochromatin regions (38, 39). Therefore, the simultaneous binding of UHRF1 to nucleosomes harboring H3K9me3 and hemimethylated DNA is the basis for the ubiquitination of histone H3. Prior studies have demonstrated that hemimethylated CpG sites located in nucleosomal linker DNA, together with dimethylated or trimethylated K9 in histone H3 marks, enhance chromatin binding and ubiquitination efficiency of UHRF1 (40, 41, 42). Interestingly, UHRF1 has a unique ubiquitin E3 ligase activity that catalyzes multiple monoubiquitination of histone H3 but not polyubiquitination (14). However, despite extensive biochemical evidence, the structural basis for how UHRF1 simultaneously engages H3K9me3 and hemimethylated CpG DNA within the context of nucleosomes remains unresolved. In particular, the mechanism by which UHRF1 coordinates multivalent binding across its domains to facilitate multiple monoubiquitination events on histone H3 tails is poorly understood.

**Figure 1:**
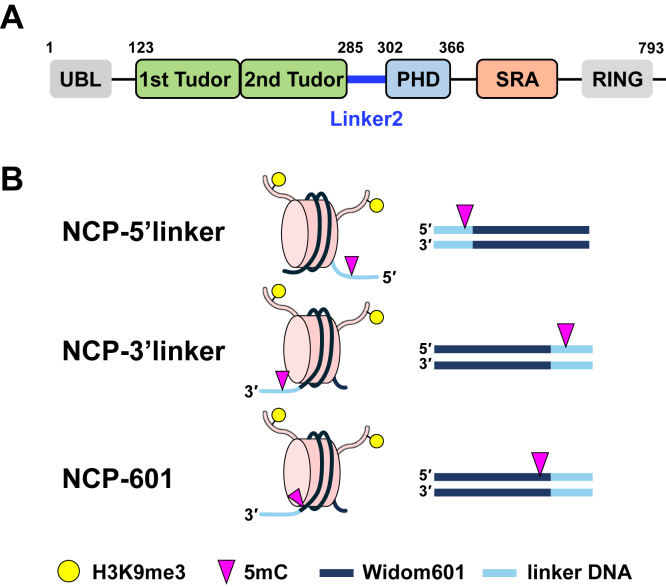
**Schematic diagrams of UHRF1 and the NCPs used in this study.***A*, schematic representation of the domain composition of UHRF1: UBL (*gray*), TTD (*green*), PHD finger (*light blue*), SRA (*orange*), and RING (*gray*). TTD consists of two Tudor domains. *B*, diagram illustrating three types of reconstructed NCPs, featuring symmetrical H3K9me3 and a single hemimethylated CpG site positioned differently. NCP, nucleosome core particle; PHD, plant homeodomain; SRA, SET and RING-associated domain; TTD, tandem Tudor domain; UBL, ubiquitin-like; UHRF1, ubiquitin-like with plant homeodomain and RING finger domain 1.

In this study, we elucidated cryo-EM structures of full-length human UHRF1 complexed with nucleosome core particles (NCPs) containing H3K9me3 and a single hemimethylated CpG site positioned at different locations. These structures revealed an unexpected interaction between the UHRF1 TTD domain and the acidic patch of the NCP. Structural analysis identified a conserved arginine-anchor (Arg-anchor) within the TTD responsible for this interaction. Biochemical assays revealed that the TTD–acidic patch interaction contributes to the efficient ubiquitination of the histone H3 tail in the NCP by UHRF1. Our data provide mechanistic insights into the binding of UHRF1 to NCP and multiple monoubiquitination of histone H3 tails.

## Results

### Properties of UHRF1 binding to NCPs harboring H3K9me3 and a single hemimethylated CpG site

**Figure 2:**
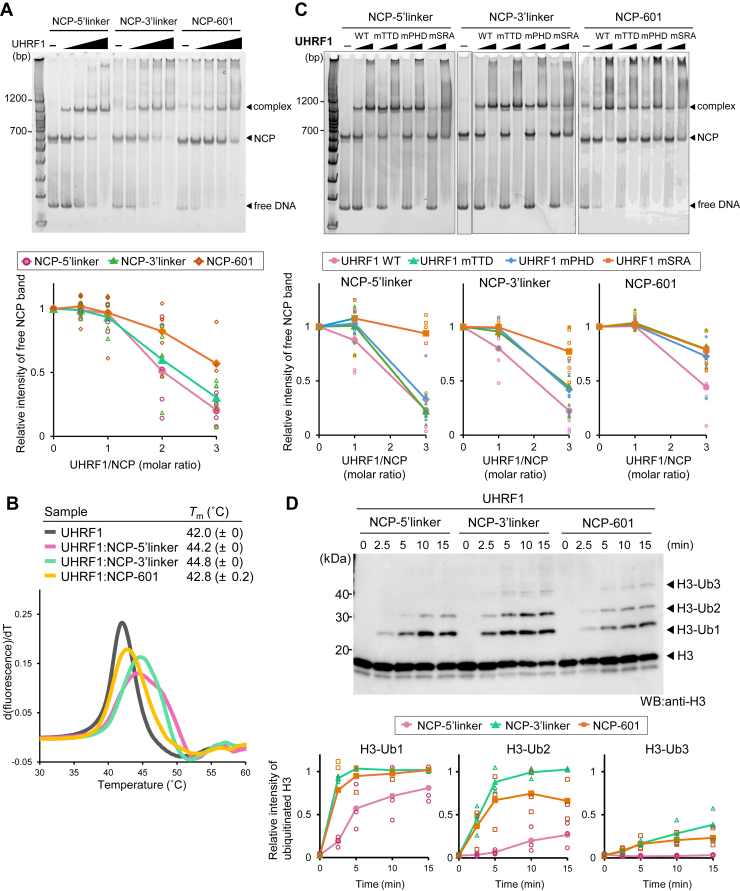
**Binding of UHRF1 to NCPs and ubiquitination of histone H3.***A*, native EMSA using UHRF1 WT and the three types of NCPs. DNA was stained using GelRed. The *bottom panel* shows the quantification of the free NCP band intensity. The intensity at 0 molar ratio of UHRF1 was set to 1.0, and relative intensities at the indicated UHRF1–NCP ratios are shown. Three independent experiments were performed. *B*, thermal stability assay of free UHRF1 (*gray*) and UHRF1 in the presence of the NCP-5′linker (*pink*), NCP-3′linker (*green*), and NCP-601 (*yellow*). Melting curves were plotted from 30 °C to 60 °C, representing average values of three independent experiments. Standard deviations are listed in the table. *C*, EMSA using UHRF1 WT and UHRF1 mutants Y188A/Y191A (mTTD), D334A/D337A (mPHD), and D469G/R491A (mSRA) in the presence of the three NCP types. DNA was stained with GelRed. The *bottom panels* show the relative intensity of free NPC band intensity at the indicated UHRF1–NCP ratios. Three independent experiments were performed. *D*, *in vitro* ubiquitination assay using UHRF1 WT and three types of NCPs. Ubiquitinated H3 was detected by Western blotting with an anti-H3 antibody. Three independent experiments were performed. The *bottom panels* show the quantification of the band intensities of H3-Ub1, H3-Ub2, and H3-Ub3. The intensities were normalized to the intensity of H3 (Ub0) at the same time point. NCP, nucleosome core particle; PHD, plant homeodomain; SRA, SET and RING-associated domain; UHRF1, ubiquitin-like with plant homeodomain and RING finger domain 1.

To evaluate the binding mode of full-length human UHRF1 to NCP, we reconstructed three types of NCPs containing both H3K9me3 and a single hemimethylated CpG site at different positions on the upper strand of DNA at the Widom 601 sequence (a nucleosome positioning sequence) (Fig. 1*B*). We named them NCP-5′linker (with a 15 bp linker DNA), NCP-3′linker (with a 12 bp linker DNA), and NCP-601 (with a 5 bp linker DNA); the NCPs harbor a single hemimethylated CpG site at the 5′-linker DNA, the 3′-linker DNA, and within the 3′-Widom 601 sequence, respectively (Figs. 1*B* and S1*A* and Table S1). In the NCP-5′linker and NCP-3′linker constructs, the hemimethylated CpG sites are located six and three bases outside the Widom 601 sequence, respectively, with the major groove facing away from the nucleosome dyad axis. In NCP-601, the CpG site is positioned nine bases inside the 3′-end of the Widom 601 sequence, with the major groove facing outward from the nucleosome core (Fig. S1*A*). These configurations assume that the Widom 601 sequence is tightly wrapped around the histone octamer, without any positional shift (Fig. S1*A*). Given that ubiquitination of histone H3 has been shown to play a role in the DNA methylation maintenance in heterochromatic late-replicating regions, we incorporated the H3K9me3 modification in the NCPs to mimic this chromatin context (6, 7). Uniform H3K9me3 modification was introduced using the H3K9-specific methyltransferase *Neurospora crassa* Dim-5 (43, 44). The NCPs contained symmetrical H3K9me3 modifications (Fig. 1*B*). The native EMSA demonstrated that UHRF1 binds to all types of NCPs independent of the position of the hemimethylated CpG site (Fig. 2*A*). The patterns of migrating bands corresponding to the UHRF1–NCP-5′linker and UHRF1–NCP-3′linker complexes were similar in the low concentration range of UHRF1 (Fig. 2*A*). In contrast, the free NCP-601 band remained at a high concentration of UHRF1, suggesting that the binding affinity of UHRF1 for NCP-601 was relatively low compared with that of other NCPs (Fig. 2*A*). Due to the differences in reconstruction efficiency among the three types of NCPs, we could not exclude the binding of UHRF1 to free DNA, particularly in NCP-5′linker, where free DNA remains (Figs. 2*A* and S1*B*). However, under these conditions, NCP-5′linker exhibited a mobility shift at a low concentration of UHRF1 compared with NCP-601, suggesting that UHRF1 binds more strongly to NCP-5′linker than to NCP-601 (Fig. 2*A*). While this observation is not strictly quantitative, it indicates a preferential binding that is unlikely to be solely explained by the presence of free DNA. Thermal stability assay also supports the EMSA results; the thermal stability of the UHRF1 bound to NCP-5′linker and NCP-3′linker (denaturation temperature [*T*_m_]: 44.2 and 44.8 °C, respectively) was higher than that bound to NCP-601 (*T*_m_: 42.8) (Figs. 2*B* and S2), implying that UHRF1 binds more stably to NCP-5′linker and NCP-3′linker than to NCP-601. To further confirm the stoichiometry of UHRF1 binding to NCPs, mass photometry measurements were performed (45). The three major peaks indicated approximately 100, 200, and 300 kDa, which corresponded to the molecular mass of UHRF1 in the free state, NCP in the free state, and UHRF1–NCP complex with a 1:1 ratio, respectively, supporting the EMSA results that the major migration band for the complex with UHRF1 indicated a 1:1 stoichiometric complex (Figs. 2*A* and S3).

It has been reported that the TTD, PHD, and SRA domains of UHRF1 simultaneously bind to the nucleosome *via* interactions with H3K9me3, ^1^ARTK^4^ sequence of the H3 N-terminal tail, and hemimethylated DNA, respectively (40, 46). To understand which domain is important for the interaction between UHRF1 and NCPs, we performed an EMSA using UHRF1 mutated critical residues for ligand recognition in the TTD (Y188A/Y191A: mTTD), PHD (D334A/D337A: mPHD), and SRA (D469G/R491A: mSRA) domains (23, 27, 47). The results showed that mutations in the SRA domain markedly reduced binding to the NCP-5′linker and NCP-3′linker (Fig. 2*C*), suggesting that SRA binding to hemimethylated DNA is more dominant than TTD–PHD binding to the histone H3 tail in interaction with NCP-5′linker and NCP-3′linker. Because NCP-601 contains only a single hemimethylated CpG site within the Widom 601 positioning sequence, the SRA domain is unable to bind effectively, thereby increasing the relative contribution of the TTD–PHD module to UHRF1–NCP interactions (Fig. 2*C*). Notably, the mechanism underlying the reduced binding of UHRF1 mSRA to NCP-601 remains unclear. Although the hemimethylated site is embedded within the NCP, potentially hindering access by the SRA domain, we cannot rule out the possibility that nonspecific interactions between the SRA domain and the linker DNA in NCP-601 contribute to the observed binding (23, 24). Taken together, these data indicated that the binding mode of UHRF1 to the NCP changed depending on the position of the hemimethylated CpG site in the NCP.

### The position of the hemimethylated CpG site regulates the ubiquitination of the histone H3 tail in the NCP

Next, we examined the ubiquitination activity of UHRF1 toward NCPs by an *in vitro* ubiquitination assay. Ubiquitinated histone H3 was detected by Western blotting with an anti-H3 antibody. Thus, our system could not detect the autoubiquitination of UHRF1 (40). The *in vitro* ubiquitination assay demonstrated that UHRF1 ubiquitinated the histone H3 tail in all types of NCPs, independent of the position of the hemimethylated CpG site (Fig. 2*D*). A similar ubiquitination property was reproduced when ubiquitin in which all lysine residues were mutated (K6, K11, K27, K29, K33, K48, and K63) to arginine residues (UbK0) were used in the *in vitro* ubiquitination assay to prevent polyubiquitination, indicating that UHRF1 catalyzes multiple monoubiquitination of the histone H3 tail in the NCP (Fig. S4). Interestingly, UHRF1 catalyzed ubiquitination at two sites on the histone H3 tail of the NCP-5′linker, although three sites were ubiquitinated in the NCP-3′linker and NCP-601 (Fig. 2*D*), suggesting that the position of the hemimethylated CpG site on the NCP affects the ubiquitination efficiency of UHRF1.

Collectively, UHRF1 catalyzes multiple monoubiquitination of histone H3 tail in the NCP. The efficiency of ubiquitination is slightly affected by the position of the hemimethylated CpG site within the NCP.

### Cryo-EM structures of UHRF1 bound to NCPs

**Figure 3:**
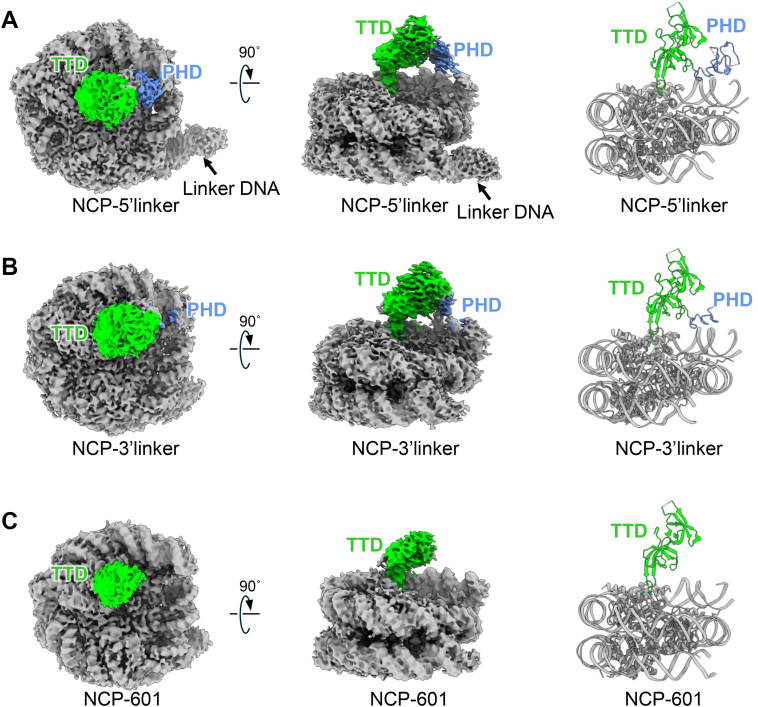
**Cryo-EM structures of UHRF1–NCPs.***A*, cryo-EM maps of UHRF1 in complex with the NCP-5′linker, (*B*) NCP-3′linker, and (*C*) NCP-601. The cryo-EM maps corresponding to the NCP, TTD, and PHD finger are denoted by *gray*, *green*, and *blue*, respectively. *Cartoon models* of the UHRF1 TTD–PHD domain (PDB ID: 3ASK) and NCPs (PDB ID: 3LZ0) are displayed on the *right side* of *A* and *B*. The *right side* of (*C*) shows the *cartoon model* of the NCP bound to the UHRF1 TTD. NCP, nucleosome core particle; PDB, Protein Data Bank; PHD, plant homeodomain; TTD, tandem Tudor domain; UHRF1, ubiquitin-like with plant homeodomain and RING finger domain 1.

To reveal the structural basis for the binding mode of UHRF1 to NCPs, we performed cryo-EM single-particle analysis of UHRF1 in complex with the NCP-5′linker, NCP-3′linker, and NCP-601 and successfully reconstructed the cryo-EM maps at 2.65, 2.88, and 2.85 Å resolution, respectively (Fig. 3, *A*–*C*, Table 1, Figs.S5–S7). We fitted the canonical NCP including the Widom 601 sequence (Protein Data Bank ID: 3LZ0) to the cryo-EM map. The histone octamer and nucleosomal DNA of the three NCP types did not undergo any structural changes upon binding of UHRF1 (48), indicating that the binding of UHRF1 did not affect the core structure of the NCP (Fig. 3, *A*–*C*). Cryo-EM densities corresponding to linker DNA in the NCP-3′linker and NCP-601 was completely invisible (Fig. 3, *B* and *C*). In contrast, the cryo-EM density of the 5′-linker DNA moiety in the NCP-5′linker was ambiguous (Fig. 3*A*). However, the map of the 5′-linker DNA exhibited a markedly low resolution, exceeding 7 Å (Fig. S8*A*), suggesting high flexibility of the linker DNA despite SRA domain binding.

**Table 1:** Cryo-EM data collection statistics

Data collection parameters	UHRF1–NCP-5′linker	UHRF1–NCP-3′linker	UHRF1–NCP-601
Electron Microscopy Data Bank	EMD-39541	EMD-39548	EMD-39550
Microscope	Krios G4 (RIKEN BDR)		
Camera	K3/BioQuantum		
Magnification	10,500×		
Voltage (kV)	300		
Electron exposure (e−/Å^2^)	59.623	59.623	58.582
Defocus range (μm)	−2.2 to −0.6		
Pixel size (Å)	0.83		
Symmetry imposed	*C*1		
Initial particle images (no.)	5,500,695	6,537,966	5,321,941
Final particle images (no.)	452,358	347,226	229,682
Map resolution (Å)	2.65	2.88	2.85
Forward scatter threshold	0.143		

Interestingly, we observed an unassigned cryo-EM density on one lateral side of the NCP disk in all NCP variants. To visualize the density more clearly, local refinement and 3D classification of the UHRF1–NCP complexes were conducted, resulting in an improved map (Fig. 3, *A*–*C*). The TTD of UHRF1 is positioned on the disk surface of the NCP according to the characteristic shape of cryo-EM density (Fig. 3, *A*–*C*). In particular, the shape of the cryo-EM density in the NCP-5′linker and NCP-3′linker is structurally consistent with the UHRF1 TTD–PHD moiety, in which TTD and PHD make dense contacts by tethering linker 2, forming a ring-shaped higher-order structure (27). We manually fitted the atomic coordinates of TTD–PHD (Protein Data Bank ID: 3ASK) to the cryo-EM density and rationally rebuilt the model of the UHRF1 TTD–PHD domains bound to the NCPs (Fig. 3, *A* and *B*). The flexible linker (∼35 residues) between the PHD finger and the SRA domain appears sufficient to spatially connect the TTD–PHD module bound on the NCP disk with the SRA domain engaging the linker DNA. In the structure of the UHRF1–NCP-601 complex, we built only a part of the second tudor moiety of the UHRF1 TTD, as the density on the NCP surface was more obscure than those of the other complexes (Fig. 3*C*). In particular, the pre-PHD moiety in the PHD finger (which is divided into pre-PHD and core-PHD moieties) is close to the DNA end of the NCP in the complex with the NCP-5′linker and NCP-3′linker (Fig. 3, *A* and *B*). The cryo-EM map also suggested a different binding angle of the UHRF1 TTD to the NCP surface; the inclination of the TTD moiety relative to the NCP surface tended to vary (Fig. S8*B*).

Taken together, the cryo-EM structures revealed an unexpected binding mode of the UHRF1 TTD to the acidic patch of the NCP and revealed that the binding mode of the TTD on the disk surface of the NCP showed variable inclination.

### Interaction between the Arg-anchor of the UHRF1 TTD and the acidic patch of the NCP

**Figure 4:**
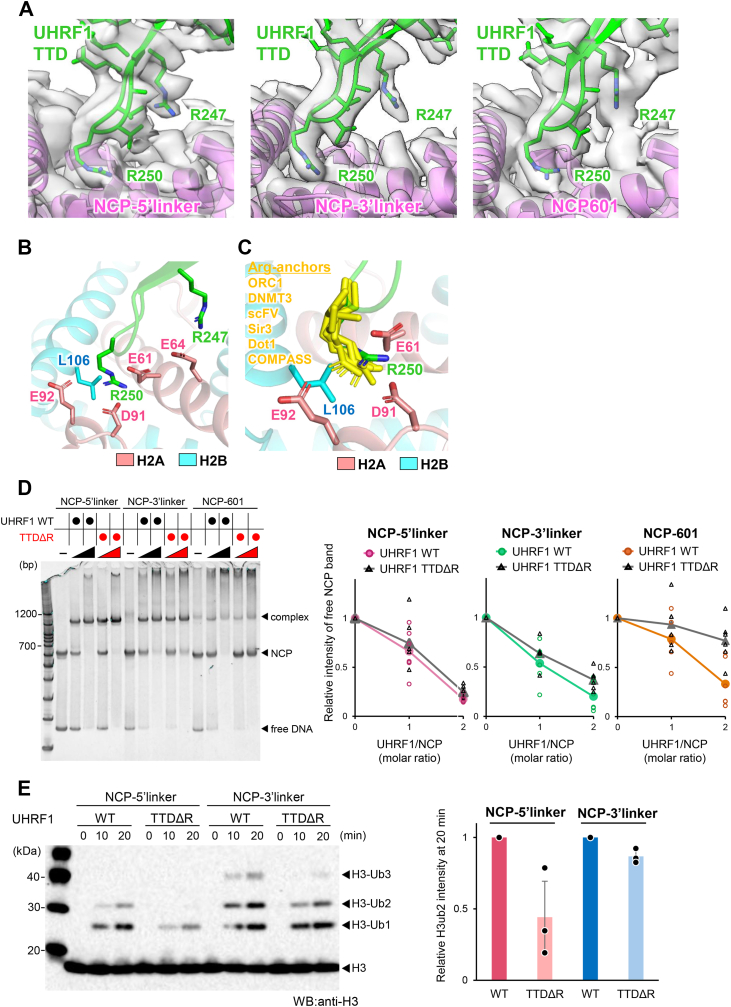
**Interaction between the Arg-anchor of the UHRF1 TTD and the acidic patch of the NCP.***A*, interaction between the Arg-anchor of the UHRF1 TTD and the acidic patch of the NCP in complex with the NCP-5′linker (*left*), NCP-3′linker (*center*), and NCP-601 (*right*). The cryo-EM map is represented as a *gray semitransparent* surface model. The UHRF1 TTD and histones are depicted as *green* and *pink cartoon* models, respectively. *B*, structure of the acidic patch bound to the Arg-anchor of UHRF1 TTD. The Arg-anchor (Arg250) of the UHRF1 TTD is indicated by a *green stick model*. The residues comprising the acidic patch, H2A (Glu61, Glu64, Asp91, and Glu92), and H2B (Leu106) are depicted as *pink* and *cyan sticks*, respectively. *C*, structural comparison of acidic patch–binding proteins. Arg-anchors from ORC1 (PDB ID: 6OM3), DNMT3 (PDB ID: 6PA7), scFV (PDB ID: 6DZT), Sir3 (PDB ID: 3TU4), Dot1 (PDB ID: 7K6Q), and COMPASS (PDB ID: 6VEN) are represented as *yellow stick models*. *D*, EMSA detecting the interaction of UHRF1 WT or UHRF1 TTDΔR (a mutant containing R247A, R250A, and R270A) with NCPs. DNA was stained with GelRed. Three independent experiments were performed. The *right panels* show the quantification of the free NCP band intensity. The intensity at 0 molar ratio of UHRF1 was set to 1.0, and relative intensities at the indicated UHRF1–NCP ratios are shown. *E*, *in vitro* ubiquitination assay using UHRF1 WT and TTDΔR. Ubiquitinated H3 was detected by Western blotting using an anti-H3 antibody. The *right panel* shows a bar graph comparing the quantified band intensities of H3ub2 in the presence of UHRF1 WT or TTDΔR. Band intensities were normalized to the WT condition, which was set as 1.0. Three independent experiments were performed. Arg-anchor, arginine-anchor; NCP, nucleosome core particle; PDB, Protein Data Bank; TTD, tandem Tudor domain; UHRF1, ubiquitin-like with plant homeodomain and RING finger domain 1.

The local resolution of cryo-EM maps around the acidic patch and arginine-rich loop of the UHRF1 TTD has a local resolution of better than 3.5 Å (Fig. S8*A*), which allows us to discuss the detailed interaction between the acidic patch and the UHRF1 TTD. Cryo-EM map showed that the arginine-rich loop of the UHRF1 TTD, comprising residues 247 to 253, contacted the acidic patch of the NCP composed of histones H2A and H2B (Fig. 4*A*), which reportedly function as a binding platform for nucleosome-binding proteins (49). The side chain of Arg250 of the UHRF1 TTD is located within hydrogen bonding and van der Waals contact distance with the side wall of the acidic patch formed by Glu61, Asp90, and Glu92 of histone H2A and Leu103 of H2B (Fig. 4, *A* and *B*). The binding mode of Arg250 in TTD is structurally conserved in other nucleosome-binding proteins, in which an Arg-anchor is inserted into the acidic patch (Fig. 4*C*) (49). In addition to Arg250, the side chain of Arg247 of the UHRF1 TTD is located within hydrogen bond distance with Asp64 of histone H2A (Fig. 4, *A* and *B*).

### TTD–acidic patch interaction facilitates ubiquitination of the histone H3 tail

To examine the functional importance of the interaction between the UHRF1 TTD and the acidic patch of the NCP, we prepared a UHRF1 mutant that replaced potential binding residues in the arginine-rich loop of the TTD with alanine residues. In addition to Arg250 and Arg247, Arg270 was selected as a potential candidate (TTDΔR) because the TTD binding angle varied relative to the NCP surface (Fig. S8, *B* and *C*). EMSA revealed that UHRF1 TTDΔR exhibited reduced binding to NCP-601 (Fig. 4*D*), suggesting that the TTD–acidic patch interaction is important when the SRA domain cannot bind to the hemimethylated CpG site within the NCP. In contrast, the UHRF1 TTDΔR bound to the NCP-5′linker and NCP-3′linker with a binding affinity similar to that of the UHRF1 WT (Fig. 4*D*), indicating that the Arg-anchor in the TTD is dispensable for binding to the NCP harboring a hemimethylated CpG site in the linker DNA.

Finally, we evaluated the ubiquitination activity of UHRF1 TTDΔR toward the histone H3 tail in NCP-5′linker and NCP-3′linker. NCP-601 was excluded from this experiment because of severe loss of UHRF1 binding (Fig. 4*D*). The ubiquitination activity of UHRF1 TTDΔR to histone H3 in the NCP-5′linker and NCP-3′linker was decreased compared with that of UHRF1 WT (Fig. 4*E*), demonstrating that the functional role of the TTD–acidic patch interaction is to facilitate the ubiquitination activity of UHRF1 to the histone H3 tail in an NCP harboring a hemimethylated CpG site in the linker DNA.

Collectively, the TTD–acidic patch interaction contributes to the efficient ubiquitination of the histone H3 tail in the NCP when the hemimethylated CpG site is positioned in the linker DNA accessible to the SRA domain.

## Discussion

Cryo-EM structures of the UHRF1–NCP complexes revealed an unexpected interaction between the UHRF1 TTD and the acidic patch on the NCP, which facilitated the ubiquitination of the histone H3 tail in NCPs harboring hemimethylated CpG site in the linker DNA. This TTD–acidic patch interaction likely stabilizes the spatial orientation of the TTD–PHD module on the nucleosome surface. This interaction was supported by AlphaFold3 (AF3) structural prediction of the UHRF1–NCP complexes (50). The TTD–acidic patch interaction was observed regardless of the presence or absence of H3K9me3 and the hemimethylated CpG site (Fig. S9), suggesting that the TTD–acidic patch interaction occurs independent of other epigenetic modifications. In this study, because of the limited cryo-EM resolution (>6 Å), the detailed binding mode of the N-terminal ^1^ARTK^4^ sequence and K9me3 of histone H3 to the TTD–PHD module could not be resolved (Fig. S8*A*). In addition, the cryo-EM densities corresponding to the UBL, SRA, and RING domains were completely invisible, preventing structural insight into their roles in nucleosome binding and histone ubiquitination. Interestingly, the pre-PHD region of UHRF1, which is unique to UHRF1 PHD finger, was positioned near the DNA end of the NCP in NCP-5′linker and NCP-3′linker. This unique structural feature, uncommon among PHD fingers, suggests an additional role of the pre-PHD in nucleosome binding or histone ubiquitination.

In this study, all experiments were conducted using the NCPs bearing H3K9me3 modification, because our aim was to investigate the binding mode of UHRF1 to late-replicating chromatin, which is enriched in H3K9me3. Consequently, we could not address the impact of the presence or absence of H3K9me3 on UHRF1 binding and NCP ubiquitination. A recent study has shown that hemimethylated DNA recruits UHRF1 to chromatin, promoting ubiquitination of H3K18, which subsequently facilitates H3K9 trimethylation by SUV39H1/2, reinforcing the heterochromatin state (51). These findings suggest that UHRF1 may interpret the modification state of H3K9 and generate distinct ubiquitination patterns to regulate chromatin dynamics. A key remaining question is how the presence or absence of H3K9me3 influences UHRF1 binding to nucleosomes and its associated ubiquitination activity.

**Figure 5:**
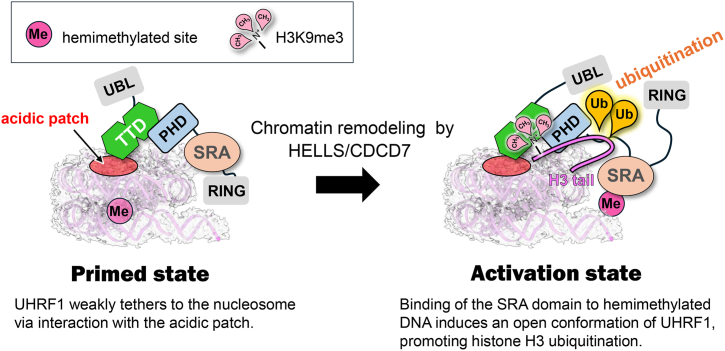
**Schematic model illustrating a potential role of the interaction between UHRF1 and the acidic patch in histone H3 ubiquitination**.

EMSA data suggest that the TTD–acidic patch interaction enables UHRF1 to bind NCP even when the SRA domain cannot access the hemimethylated CpG sites embedded within the nucleosomal core, such as those found in heterochromatic regions. AF3 structural predictions suggest that the TTD–acidic patch interaction occurs independently of the position of hemimethylated site (Fig. S9). One possible role of the TTD–acidic patch interaction is to provide weak tethering of UHRF1 to chromatin. Chromatin remodeling by HELLS–CDCA7 complex alters the position of hemimethylated site from within the nucleosome to linker DNA regions (8, 52, 53, 54, 55, 56, 57, 58), thereby allowing the SRA domain to access to the hemimethylated DNA and promoting strong binding of UHRF1 to nucleosomes. Binding of hemimethylated DNA to the SRA domain is known to induce an open conformation of UHRF1, enhancing its E3 ligase activity toward histone H3 (46, 59). Therefore, the TTD–acidic patch interaction may serve to establish a “primed” state that subsequently facilitates strong binding of UHRF1 to nucleosomes, enabling efficient ubiquitination in heterochromatic regions (Fig. 5).

Our findings demonstrated that the binding properties and ubiquitination activity of UHRF1 differed depending on the position of the hemimethylated CpG site in the NCP. However, the reason for these differences remains unclear, as the major grooves of the CpG sites in both NCP-5′linker and NCP-3′linker are equally exposed. Decreasing the flexibility of UHRF1 through chemical crosslinking may help elucidate the contribution of other UHRF1 domains to nucleosome binding and histone H3 ubiquitination. In addition, improving the resolution of the cryo-EM map will be essential to address the unresolved questions raised by this study. UHRF1 reportedly binds to the pericentromeric region including heterochromatin (60, 61). Future studies focusing on UHRF1 interactions with dinucleosomes or higher-order chromatin structures, including those with various epigenetic modifications, will provide further mechanistic insights into UHRF1-mediated chromatin regulation and histone ubiquitination.

## Experimental procedures

### Protein purification and mutagenesis

Full-length human UHRF1 complementary DNA was amplified by PCR and subcloned into the pGEX6P-1 plasmid (Cytiva). *Escherichia coli* Rosetta 2(DE3) cells were transformed with the plasmid. *E. coli* cells were precultured with 200 ml of LB medium containing 50 μg/ml ampicillin and 34 μg/ml chloramphenicol at 37 °C until saturation. The cells were then transferred to 2 l of LB medium and incubated at 37 °C. When the absorbance reached 0.7, 0.2 mM IPTG was added to the medium, and the cells were further incubated for 20 h at 15 °C. The cells were collected by centrifugation, resuspended in lysis buffer (40 mM Tris–HCl [pH 8.0], 300 mM NaCl, 10% glycerol, 30 μM zinc acetate, 0.5 mM Tris(2-carboxyethyl)phosphine hydrochloride, and protease inhibitor cocktail [Nacalai]), and sonicated on ice (cycle: 5 s on, 30 s rest, total on time: 6 min). After centrifugation at 19,000 rpm for 40 min at 4 °C (BECKMAN COULTER; Avanti J-26S, JA-20 rotor), the supernatant was loaded three times onto a GS4B column (Cytiva). After washing the column using 50 ml of wash buffer (20 mM Tris–HCl [pH 8.0], 1 M NaCl, 10% glycerol, 30 μM zinc acetate, and 0.5 mM Tris(2-carboxyethyl)phosphine hydrochloride) and lysis buffer, glutathione-*S*-transferase (GST)-UHRF1 was eluted from the column using 50 ml elution buffer (50 mM Tris–HCl [pH 8.0], 300 mM NaCl, 10% glycerol, 1 mM DTT, and 20 mM reduced glutathione). After the GST tag was removed using GST-PreScission protease, the sample was loaded onto HiTrap Heparin HP column (Cytiva) equilibrated with buffer A (20 mM Tris–HCl [pH 8.0], 50 mM NaCl, 0.5 mM DTT, 10% glycerol, and 10 μM zinc acetate), and the sample was eluted with a linear gradient of 5% to 40% buffer B (20 mM Tris–HCl [pH 8.0], 2 M NaCl, 0.5 mM DTT, 10% glycerol, and 10 μM zinc acetate) to a final volume of 100 ml. Purified UHRF1 was loaded onto a GS4FF affinity column (Cytiva) to remove the GST. The unbound fraction was concentrated using a 50 kDa molecular weight cutoff Amicon concentrator (Merck Millipore) and loaded onto a HiLoad 26/600 Superdex 200 (Cytiva) equilibrated with a buffer (10 mM Tris–HCl [pH 7.5], 500 mM NaCl, 1 mM DTT, 10% glycerol, and 10 μM zinc acetate). The eluted sample was then concentrated, frozen in liquid nitrogen, and stored at −80 °C.

The UHRF1 mutants, Y188A/Y191A, D334A/D337A, D469G/R491A, and R247A/R250A/R270A, were prepared by PCR-based mutagenesis and purified using the same method as the UHRF1 WT.

### Histone purification

Recombinant human histones were prepared as described previously (48, 62). Histones H2A, H2B, H3.1, and H4 were expressed in *E*. *coli* BL21 (DE3). *E. coli* cells were precultured with 200 ml of LB medium at 37 °C until saturation. The cells were then transferred to 2 l of LB medium and incubated at 37 °C. When the absorbance reached 0.7, 0.2 mM IPTG was added to the medium, and the cells were further incubated for 4 h at 37 °C. The cells were collected by centrifugation, suspended in wash buffer (50 mM Tris–HCl [pH 7.5], 100 mM NaCl, 1 mM PMSF, and 1 mM 2-mercaptoethanol [2-ME]), and sonicated on ice (cycle: 10 s on, 1 min rest, and total on time: 5 min). After centrifugation at 19,000 rpm for 40 min at 4 °C, the pellet was resuspended in a wash buffer containing 1% Triton X-100 and centrifuged again at 19,000 rpm for 20 min at 4 °C. The resuspension and centrifugation processes were repeated using wash buffer that did not contain 1% Triton X-100. After centrifugation, the pellet was resuspended in dimethyl sulfoxide (1 ml/6 l scale culture) and incubated at room temperature for 30 min. Unfolding buffer (6 M guanidinium–HCl, 50 mM Tris–HCl [pH 7.5], and 5 mM DTT) was added slowly to the incubated sample, which was then resuspended and sonicated on ice (cycle: 3 s on, 10 s rest, and total on time: 3 min). The sonicated samples were rotated at 4 °C for 1 h. After centrifugation at 19,000 rpm for 30 min at 20 °C, the supernatant was loaded onto HiLoad 26/600 Superdex 75 (Cytiva) equilibrated with buffer (7 M urea, 20 mM sodium acetate [pH 5.2], 5 mM 2-ME, and 1 M NaCl). The eluted sample was concentrated and dialyzed with 2 l of 1 mM DTT for 4 h at 4 °C using a 3.5 kDa cutoff dialysis membrane (BioDesign, Inc) at least three times. The dialyzed samples were frozen in liquid nitrogen and lyophilized.

The lyophilized histone proteins were suspended in SP A buffer (7 M urea, 20 mM sodium acetate [pH 5.2], 200 mM NaCl, 5 mM 2-ME, and 1 mM EDTA) and loaded onto a HiTrap SP HP column (Cytiva) equilibrated with SP buffer A. Samples were eluted with a linear gradient of 0% to 40% SP buffer B (7 M urea, 20 mM sodium acetate [pH 5.2], 2 M NaCl, 5 mM 2-ME, and 1 mM EDTA) for a final volume of 100 ml. The eluted sample was concentrated, frozen in liquid nitrogen, lyophilized, and stored at −80 °C.

### K9me3 modification in histone H3

Histone H3 powder was dissolved in a methylation buffer (50 mM Tris–HCl [pH 9.0], 2 mM DTT, and 10 μM zinc acetate). Trimethylation of K9 on histone H3 was introduced using in-house purified 5 μM *N. crassa* Dim-5, an H3K9-specific methyltransferase, and 10 mM *S*-adenosylmethionine at 15 °C for 3 h. Uniform trimethylation of histone H3 was confirmed using MALDI TOF-MS, and the reaction product was purified using HiTrap SP HP. The purified sample was concentrated, frozen using liquid nitrogen, lyophilized, and stored at −80 °C.

### Octamer preparation

Lyophilized histone proteins were resuspended in unfolding buffer and mixed in equal stoichiometry to reach a total concentration of 1.5 mg/ml. The sample was dialyzed three times with 500 ml of refolding buffer (20 mM Tris–HCl [pH 7.5], 2 M NaCl, 1 mM DTT, and 1 mM EDTA) at 4 °C for 4 h using an 8 kDa cutoff dialysis membrane (BioDesign, Inc). The sample was concentrated and loaded onto a HiLoad 16/600 Superdex 200 column equilibrated with the refolding buffer. The eluted sample was concentrated, frozen in liquid nitrogen, and stored at −80 °C.

### DNA preparation

All DNA sequences, including a single hemimethylated CpG site, were based on the Widom 601 nucleosome positioning sequence (63). For the preparation of DNA with a single hemimethylated CpG site at the 5′-linker DNA, the Widom 601 sequence was amplified using primers (Table S1). DNA was purified using HiTrap Q HP (Cytiva) equilibrated with Q buffer A (20 mM Tris–HCl [pH 7.0], and 400 mM NaCl). The DNA was eluted with a linear gradient of 30% to 80% Q buffer B (20 mM Tris–HCl [pH 7.0] and 1 M NaCl), giving a final volume of 80 ml. The buffer was removed using ethanol precipitation, and the purified DNA was stored at −20 °C.

To prepare DNA with a single hemimethylated CpG site in the 3′-linker and nucleosomal DNA, the Widom 601 sequence was amplified with a BsmBI site in the 3′-region (Table S1) and purified using the same method described above. The GAGACG sequence in DNAs was digested using BsmBI restriction enzyme (10 μl for 0.2 mg of DNA; New England Biolabs). The DNAs were digested with Q buffer-A (20 mM Tris–HCl [pH 7.0], and 400 mM NaCl) and purified using a HiLoad 26/600 Superdex 200 equilibrated with Q buffer A. Five equimolar synthetic oligonucleotides, including a hemimethylated CpG site (Table S1), were mixed with the purified DNA. The mixture was ligated using T4 DNA Ligase (1 μl for 50 μg of DNA; TAKARA). The DNA product was purified using HiTrap Q HP with a linear gradient of 30% to 80% Q buffer B in a volume of 80 ml. The resulting DNA product was precipitated with ethanol and stored at −20 °C.

### NCP reconstruction

NCP reconstruction was performed as previously described (48, 62). The histone octamer and each DNA were mixed in a 1:1 stoichiometry to a concentration of 0.7 mg/ml using 4 M KCl, ultimately reaching a final concentration of 2 M KCl. 5′linker-DNA, 3′linker-DNA, and 601-DNA were used for NCP reconstruction and were designated as NCP-5′linker, NCP-3′linker, and NCP-601, respectively. The solution was incubated on ice for 30 min and dialyzed with 400 ml of refolding high buffer (10 mM Hepes [pH 7.5], 2 M KCl, 1 mM EDTA, and 1 mM DTT) for 4 h at 4 °C using an 8 kDa cutoff dialysis membrane. The NCPs were reconstructed using the salt dialysis method. The buffer was exchanged with 1600 ml of a refolding low buffer (10 mM Hepes [pH 7.5], 0.25 M KCl, 1 mM EDTA, 1 mM DTT) for 36 h at 4 °C. Reconstructed NCPs were dialyzed with refolding low buffer for 4 h at 4 °C, heated at 55 °C for 2 h, and purified using HiTrap Q HP equilibrated with Q buffer-A. The sample was eluted with a linear gradient of 30% to 55% Q buffer B for a final volume of 90 ml. The purified NCP was concentrated to 200 μl using an Amicon Ultra 30 KDa cutoff (Merck Millipore) and dialyzed with a storage buffer (20 mM Tris–HCl [pH 7.5], 1 mM DTT, and 5% glycerol) and an 8 kDa cutoff Mini dialysis kit (Cytiva) for 15 h at 4 °C. NCPs were stored at −80 °C.

### *In vitro* ubiquitination assay

The recombinant ubiquitin E1 enzyme, mouse ubiquitin-activating enzyme (mUBA1), E2 enzyme (UBE2D3), and ubiquitin were prepared in-house as previously reported (6, 64). Overall, 0.1 μM NCP, 0.2 or 0.3 μM UHRF1 (E3), 0.2 μM mUBA1, 4 μM UBE2D3, and 20 μM ubiquitin were mixed in a 30 μl reaction solution containing 50 mM Tris–HCl (pH 8.0), 50 mM NaCl, 5 mM MgCl_2_, 0.5% Triton X-100, 8 mM ATP, and 2 mM DTT. The reaction mixture was incubated for 0 to 20 min at 30 °C. The sample was denatured at the indicated times using 3× SDS sample buffer and heated at 95 °C for 2 min. Histone H3 ubiquitination was detected by Western blotting with an anti–histone H3 antibody (Abcam; #ab1791). The specificity of antibodies was verified based on signal size and the absence of nonspecific bands. ChemiDoc XRS system (Bio-Rad) was used for band detection. At least three independent experiments were performed in the ubiquitination assay to confirm reproducibility. Quantification of band intensity was performed using ImageJ software (https://imagej.net/ij/). For Figure 2*D*, the band intensities of H3-Ub1, H3-Ub2, and H3-Ub3 were normalized by dividing them by the band intensity of unmodified H3 at the same time point. For Figure 4*E*, the band intensities were normalized to the WT condition, which was set to 1.0 to compare the band intensity in the presence of the mutant.

### Electrophoresis mobility shift assay

NCP (0.1 μM) was incubated with increasing concentrations of WT and indicated mutant UHRF1. The molar ratios of NCP to UHRF1 WT were 1:0, 1:0.5, 1:1, 1:2, and 1:3 (Fig. 2*A*), whereas the ratios for NCP to UHRF1 mutants were 1:0, 1:1, and 1:3. A total of 10 μl of the reaction mixture in EMSA buffer (20 mM Tris–HCl [pH 7.5], 150 mM NaCl, 1 mM DTT, and 10% glycerol) was incubated on ice for 30 min. Electrophoresis was performed using 7.5% SuperSep (FUJIFILM Wako) at a constant current of 10 mA for 90 min at 4 °C. DNA in the gel was detected using GelRed (FUJIFILM Wako) and ChemiDoc XRS system. At least three independent experiments were conducted using the EMSA. Quantification of band intensity was performed using ImageJ software.

### Thermal stability assay

The change in the denaturation temperature of the UHRF1–NCP complex was evaluated by a thermal stability assay using SYPRO Orange (Thermo Fisher Scientific). The assay was performed in 20 μl of 0.2 mg/ml protein at a concentration, dissolved in a buffer (20 mM Tris–HCl [pH 7.5], 150 mM NaCl, and 1 mM DTT). To detect the denaturation temperature, the UHRF1–NCP complex was heated from 25 °C to 90 °C in increments of 0.2 °C every 10 s using a temperature gradient. Fluorescence intensity was measured using a CFX Connect Real-Time System (Bio-Rad) and a 96-well PCR plate (Bio-Rad). The measured fluorescence data were normalized to (*F*(T) - *F*_min_)/(*F*_max_ – *F*_min_), where *F*(T) is the fluorescence intensity at a particular temperature, *F*_max_ is the maximum fluorescence intensity, and *F*_min_ is the minimum fluorescence intensity (65). Three independent experiments were performed using the thermal stability assay.

### Mass photometry analysis

Mass photometry measurements were performed on cleaned glass cover slips and recorded on a mass photometer (Two^MP^; Refeyn Ltd). Mass calibration for mass photometry was performed using a 40-fold dilution of bovine serum albumin, human immunoglobulin G, and thyroglobulin (MARUWA foods). The resulting peaks in the mass photometry spectrum corresponding to bovine serum albumin (66 kDa), human immunoglobulin G (150 kDa), and thyroglobulin (660 kDa) were fitted with Gaussian functions to obtain their centroids, which were used to linearly calibrate all reported spectra for that specific slide. PBS (10 μl) was then loaded onto the sample cassette. UHRF1 and the three types of NCPs were mixed at a UHRF1–NCP ratio of 1:4 with buffer (20 mM Tris–HCl [pH 7.0], 150 mM NaCl), and the mixtures were incubated on ice for 30 min. A 10 μl droplet of UHRF1–NCP sample was applied onto a 10 μl droplet to a final concentration of 25 nM in 20 mM Tris–HCl (pH 7.0), and 150 mM NaCl. Mass photometry videos of both the calibration and the UHRF1–NCP solutions were recorded for 1 min and analyzed using DiscoverMP 2.3.0 (Refeyn). Three independent experiments were performed.

### Cryo-EM sample preparation and data collection

UHRF1 and three types of NCPs were mixed in a 1:4 (NCP-5′linker–UHRF1), 1:2 (NCP-3′linker–UHRF1), and 1:4 (NCP-601–UHRF1) molar ratio and adjusted to a final concentration of 2.2 μM in a cryo-buffer (20 mM Tris–HCl [pH 7.5], 50 mM [or 150 mM] NaCl, and 1 mM DTT). The NCP-3′linker–UHRF1 mixture showed good dispersion of the particles in 150 mM NaCl. The mixture was incubated on ice for 30 min. The samples were then centrifuged at 15,000 rpm for 1 min at 4 °C. The solution (3 μl) was applied to glow-discharged holey carbon grids (Quantifoil Cu R1.2/1.3, 300 mesh), which were plunge-frozen in liquid ethane using a Vitrobot Mark Ⅳ (Thermo Fisher Scientific) at 4 °C and 100% humidity. The UHRF1–NCP-5′linker and UHRF1–NCP-3′linker complexes were blotted for 3 s with a waiting time of 3 s at a blotting force of −10, and the UHRF1–NCP-601 complex was blotted for 3 s with a waiting time of 3 s at a blotting force of 0 before freezing in liquid ethane. Data were collected at RIKEN BDR on a 300 kV Krios G4 (Thermo Fisher Scientific) using a BioQuantum K3 detector (Gatan) with a BioQuantum energy filter. A total of 12,001 (UHRF1–NCP-5′linker complex), 14,000 (UHRF1–NCP-3′linker complex), and 8352 (UHRF1–NCP-601 complex) movies were recorded at a nominal magnification of 105,000× with a pixel size of 0.83 Å, with the defocus range from −2.2 to −0.6 μm and the dose rate of 1.24 electrons/A^2^ per frame (UHRF1–NCP-5′linker and UHRF1–NCP-3′linker complexes), and 1.22 electrons/A^2^ per frame (UHRF1–NCP-601 complex).

### Cryo-EM data processing

Cryo-EM data for the UHRF1–NCP complexes were processed with CryoSPARC (version 4.2.1 and version 4.4.0) (66). The movies were motion-corrected, and defocus values were estimated from contrast transfer function (67). Micrographs with resolutions under 8 Å contrast transfer function were cutoff using Curate Exposures, and the particles were automatically picked using Blob Picker. A total of 4,205,473 particles from 11,637 micrographs, 5,476,617 particles from 13,685 micrographs, and 3,960,102 particles from 7851 micrographs were obtained for the UHRF1-NCP-5′linker, UHRF1-NCP-3′linker, and UHRF1-NCP-601, respectively. These particles were extracted using extract from micrographs, in which particles were classified by several rounds of 2D classification divided by particle orientation. An initial model of the NCP and junk particle model was created using *ab initio* reconstruction, which was used as a template for heterogeneous refinement. Particle selection using 2D classification and heterogeneous refinement were repeated several times. The selected suitable particles used for reconstructing the 3D volume by nonuniform refinement, and the focused mask corresponding around the UHRF1 TTD was created using ChimeraX (version 1.5) (68), which was used for further classification by 3D variability analysis and visualized using 3D variability display (69). Only particles that showed the UHRF1 TTD were collected, with 1,269,411, 1,856,468, and 1,048,928 particles for the UHRF1–NCP-5′linker, UHRF1–NCP-3′linker, and UHRF1–NCP-601 complexes, respectively. The collected particles were further sorted using heterogeneous refinement and 2D classification. The mask surrounding the UHRF1 TTD (TTD-mask) was created again. The cryo-EM structure of the UHRF1–NCP-601 complex was obtained using the aforementioned process with 229,682 particles. The UHRF1–NCP-5′linker and UHRF1–NCP-3′linker were further processed as follows. With regard to signals that were not surrounded by the mask in the full volume of UHRF1–NCP complex were removed using particle subtraction. The subtracted particles and the TTD-mask were used for 3D classification to categorize them according to the TTD orientation relative to the NCP. The 3D classification was repeated several times, and several suitable classes were selected, which were further curated by heterogeneous refinement. The selected suitable classes included 452,358, 347,226, and 229,682 particles for the UHRF1–NCP-5′linker, UHRF1–NCP-3′linker, and UHRF1–NCP-601 complexes, respectively. Finally, the 3D volume was reconstructed using selected particles and sharpened by using a *B*-factor of −65.

The figures were generated using ChimeraX and PyMOL (http://www.pymol.org). AF3 structural predictions were conducted using the AF Server (https://golgi.sandbox.google.com/).

## Data availability

The cryo-EM density maps of UHRF1 in complex with the NCP-5′linker, NCP-3′linker, and NCP-601 were deposited in the Electron Microscopy Data Bank (www.ebi.ac.uk/pdbe/emdb/) under accession codes EMD-39541, EMD-39548, and EMD-39550, respectively.

## Supporting information

This article contains supporting information.

## Conflict of interest

The authors declare that they have no conflicts of interest with the contents of this article.
