# Supplementary material for: Structural basis for E3 ubiquitin ligase UHRF1 binding to nucleosome core particle and histone H3 ubiquitination
Source: J Biol Chem. 2025 Nov 4;301(12):110894. doi: 10.1016/j.jbc.2025.110894 (PMC12704277; doi:10.1016/j.jbc.2025.110894)
Supplement: Supplementary Figures and Table [file mmc1.pdf]

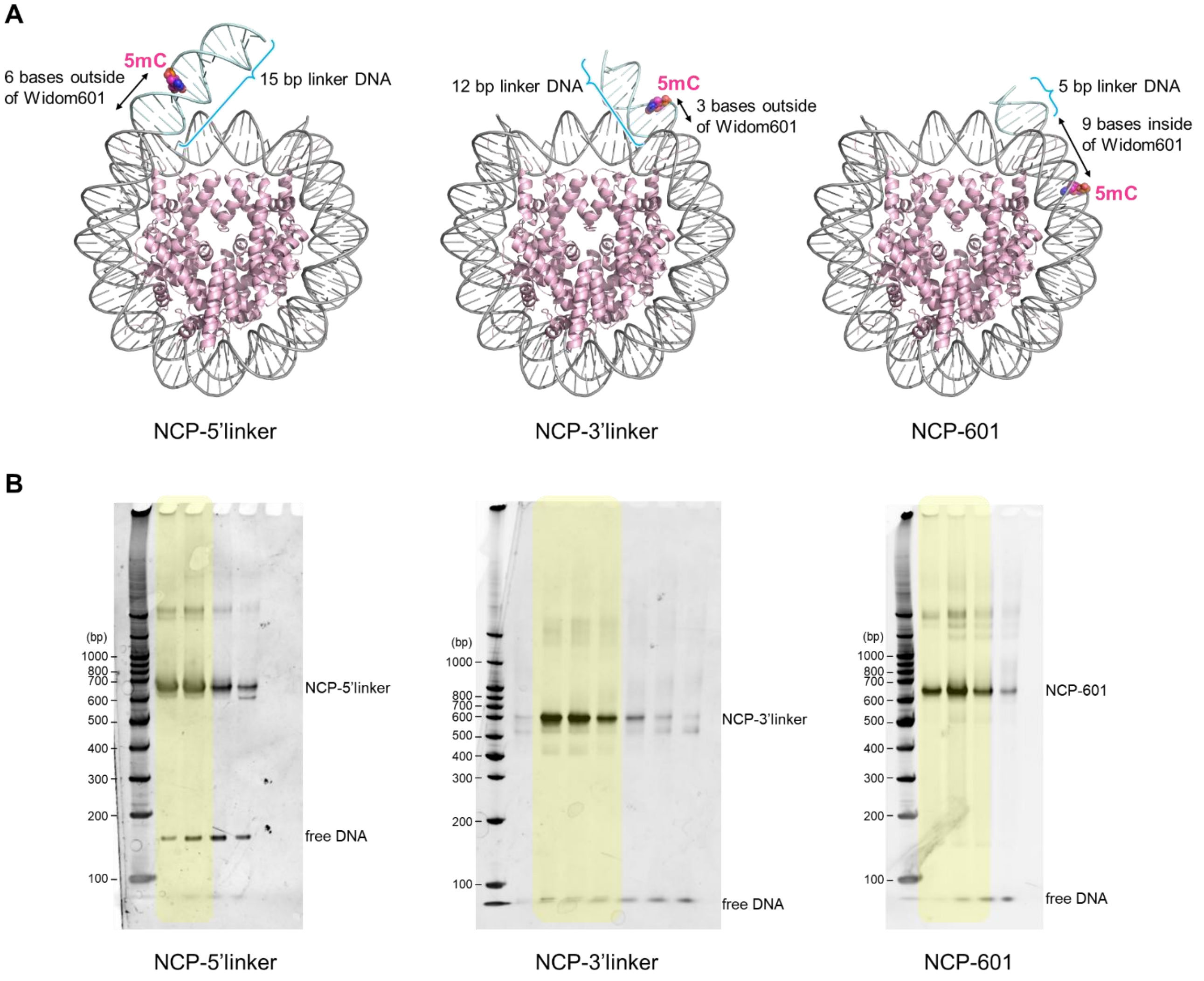

Supplementary Figure S1. Schematic representation and purity assessment of NCPs.

(A) Structural model of NCPs used in this study. The Widom 601 sequence and linker DNA are shown as gray and pale cyan cartoon representations, respectively. 5-methylcytosine (5mC) in hemimethylated site is depicted as magenta sphere. The histone octamer is depicted as a pink cartoon model with histone tails removed for clarity. **(B)** Native-PAGE analysis of NCPs purified by anion-exchange chromatography. DNA was detected using GelRed^{TM}. Fractions highlighted in light yellow were pooled, concentrated, and subsequently used for biochemical assays and cryo-EM analysis.

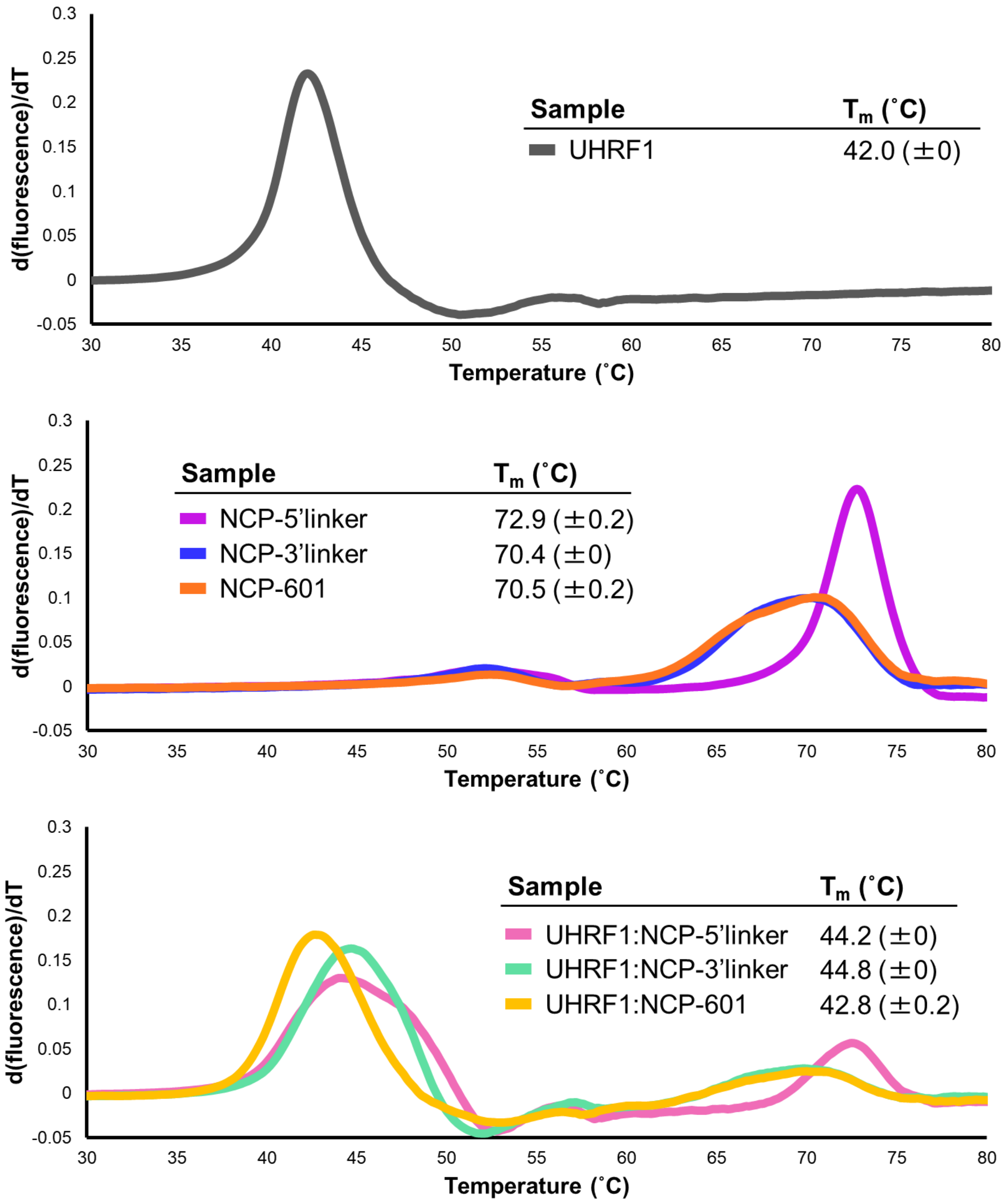

Supplementary Figure S2. Thermal stability assay

Melting curves of UHRF1 and NCPs. The upper panel shows the melting curve of free UHRF1 (gray). The middle panel displays the melting curves of NCP-5'linker (magenta), NCP-3'linker (blue), and NCP-601 (orange). The lower panel presents melting curves of UHRF1 bound to NCP-5'linker (pink), NCP-3'linker (green), and NCP-601 (yellow). All curves are plotted from 30 to 80°C, including the denaturation points of UHRF1 and histones. Three independent experiments were performed.

A

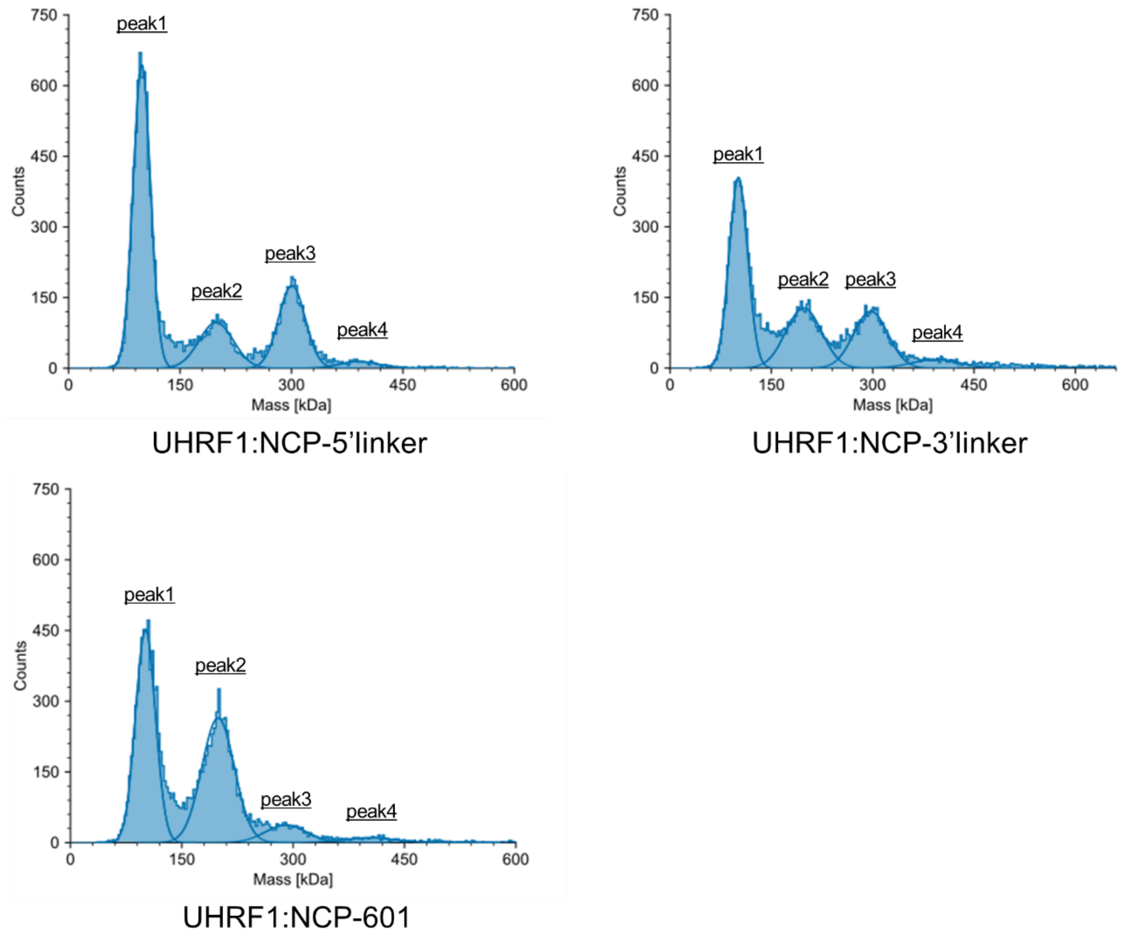

B

UHRF1:NCP-5’linker	Peak1	Peak2	Peak3	Peak4
**Sample**	UHRF1	NCP-5’linker	NCP-5’linker:UHRF1	NCP-5’linker:2UHRF1
**Molecular weight (kDa)**	99 (±11.8)	191 (±23)	301 (±17.9)	391 (±26)
**Count**	6792	2002	2816	338

UHRF1:NCP-3’linker	Peak1	Peak2	Peak3	Peak4
**Sample**	UHRF1	NCP-3’linker	NCP-3’linker:UHRF1	NCP-3’linker:2UHRF1
**Molecular weight (kDa)**	101 (±13.8)	198 (±26)	297 (±25)	394 (±35)
**Count**	4966	3019	2734	580

UHRF1:NCP-601	Peak1	Peak2	Peak3	Peak4
**Sample**	UHRF1	NCP-601	NCP-601:UHRF1	NCP-601:2UHRF1
**Molecular weight (kDa)**	101 (±13.4)	199 (±22)	290 (±28)	404 (±35)
**Count**	5420	5179	930	327

Supplementary Figure S3. Mass photometry

(A) Mass distribution of UHRF1 mixed with NCP-5’linker, NCP-3’linker and NCP-601. **(B)** Summary of the peaks resulting from mass photometry. Three independent experiments were performed.

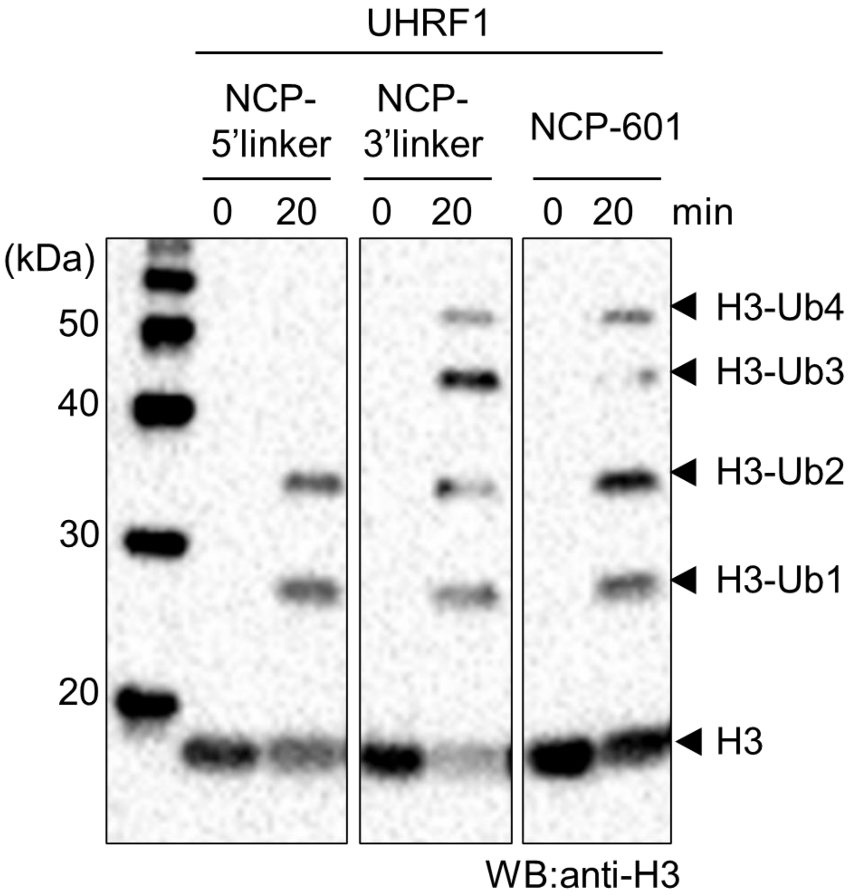

Supplementary Figure S4. *In vitro* ubiquitination assay

In vitro ubiquitination assay using UHRF1 wild-type and three types of NCPs, in which seven lysine residues (K6, K11, K27, K29, K33, K48, and K63) in ubiquitin are replaced with arginine residues. Ubiquitinated H3 was detected by western blotting (WB) using an anti-H3 antibody. Three independent experiments were performed.

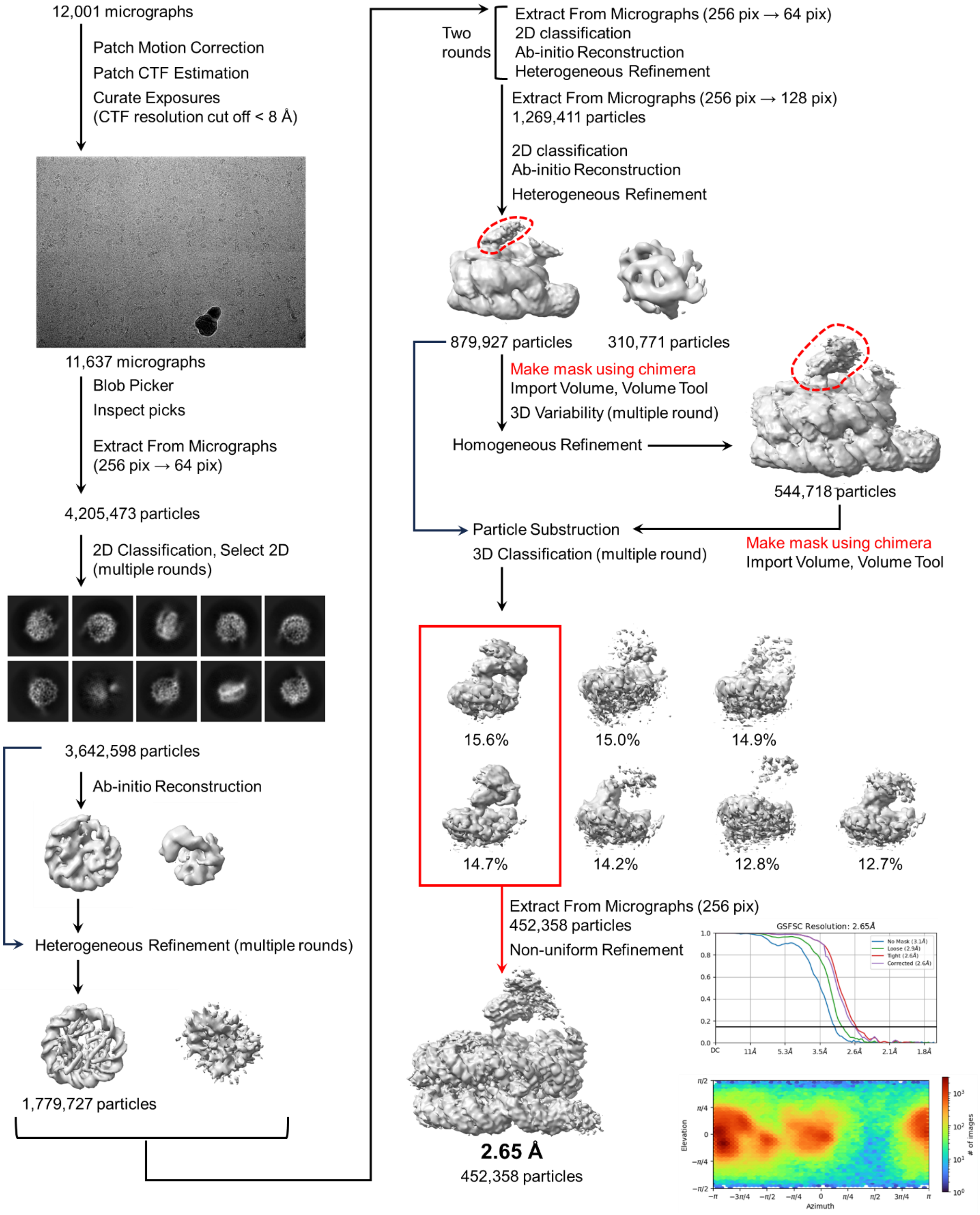

Supplementary Figure S5. Workflow of cryo-EM single-particle analysis of the UHRF1:NCP-5'linker complex. The orientation distribution of the refined particles and the Fourier shell correlation (FSC) curve are shown. The map resolution is reported at an FSC threshold of 0.143.

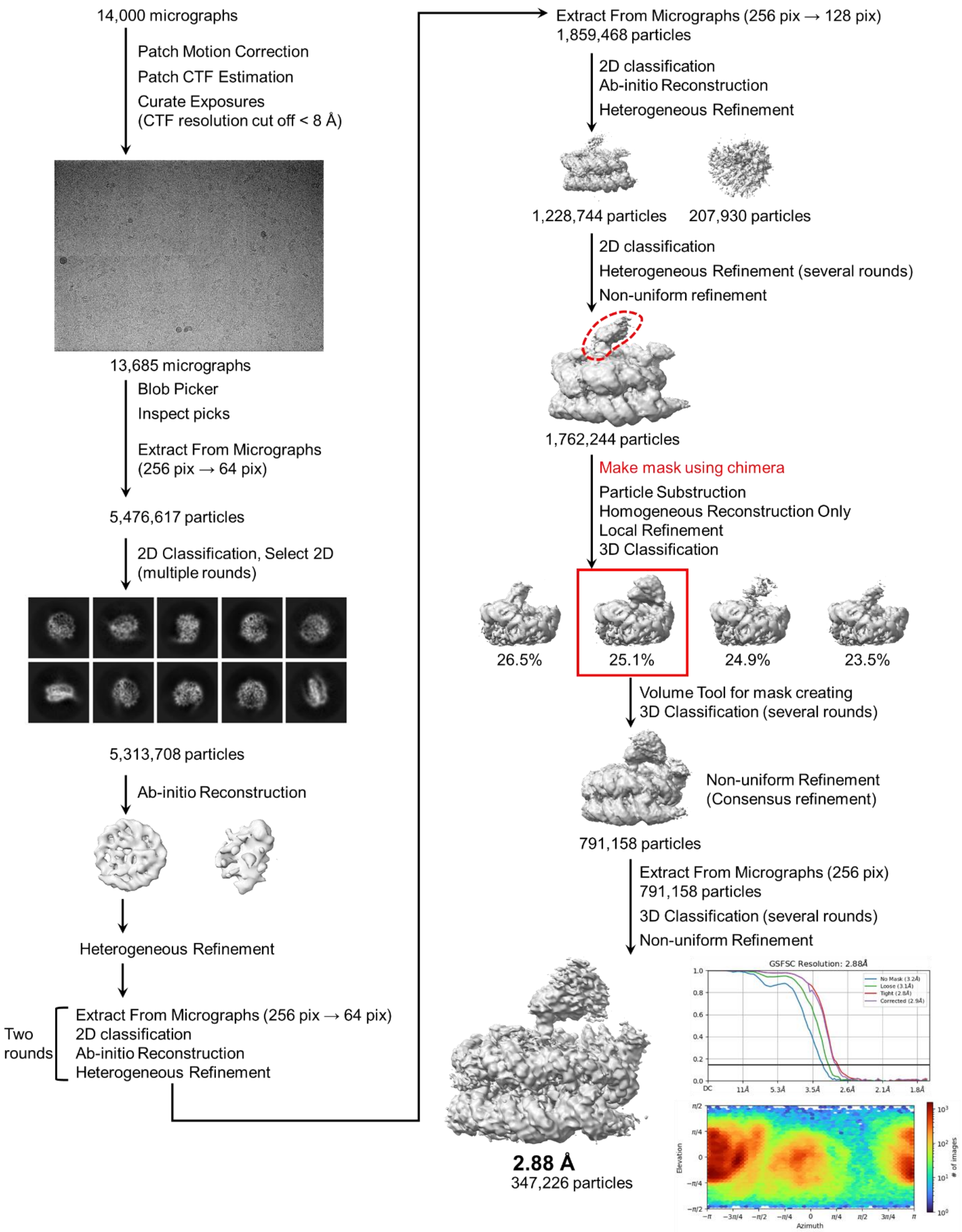

Supplementary Figure S6. Workflow of cryo-EM single-particle analysis of the UHRF1:NCP-3'linker complex. The orientation distribution of the refined particles and the FSC curve are shown. The map resolution is reported at an FSC threshold of 0.143.

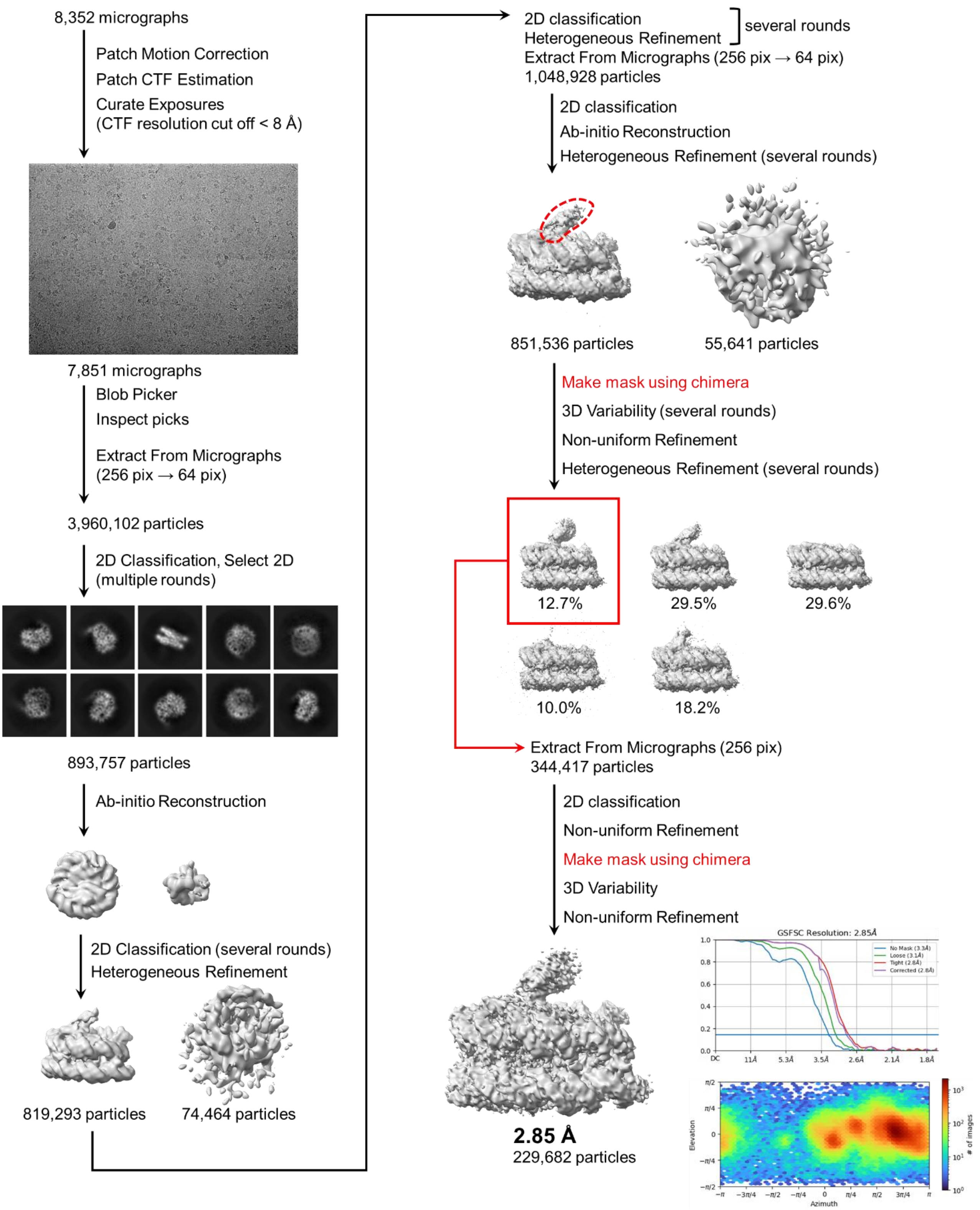

Supplementary Figure S7. Workflow of cryo-EM single-particle analysis of the UHRF1:NCP-601 complex. The orientation distribution of the refined particles and the FSC curve are shown. The map resolution is reported at an FSC threshold of 0.143.

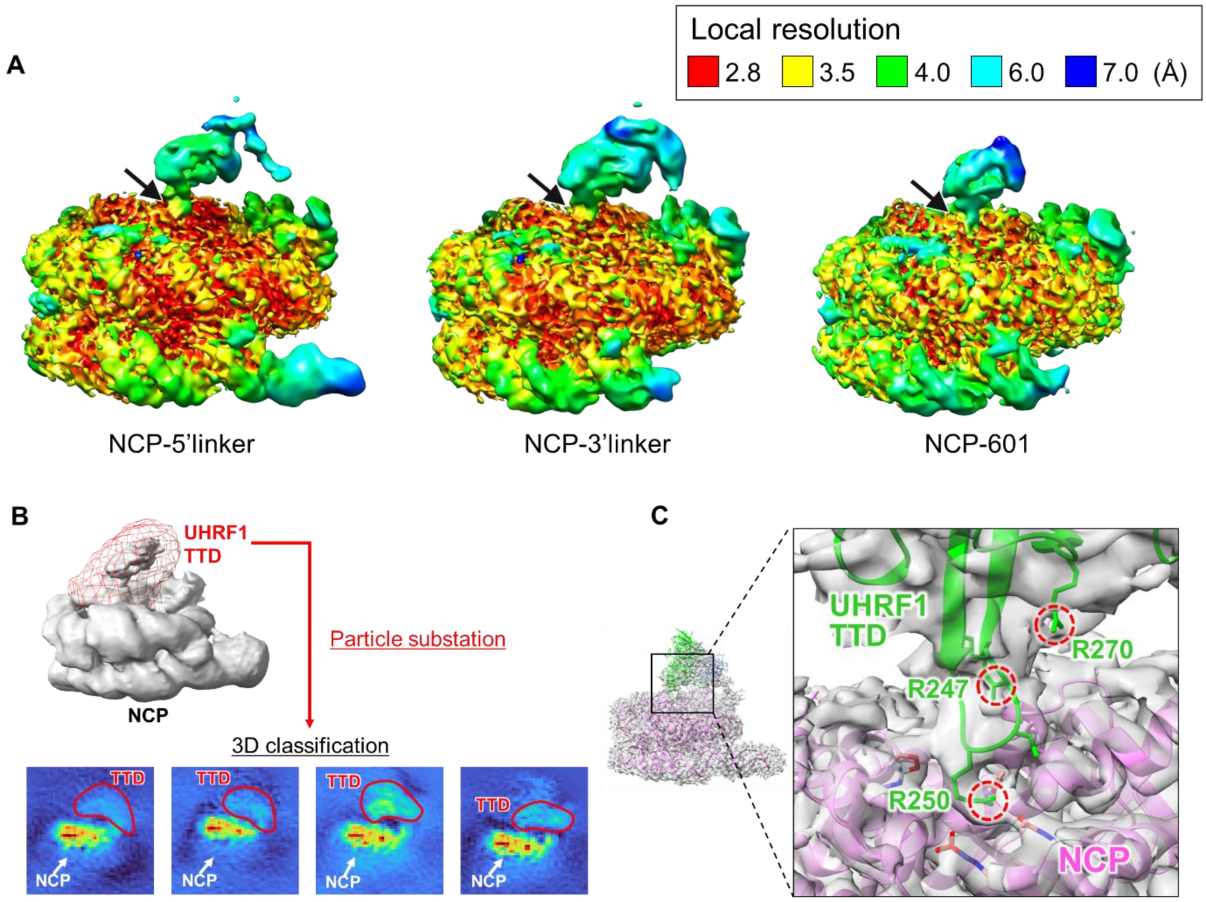

Supplementary Figure S8. Cryo-EM analysis

(A) Local resolution map of UHRF1 bound to the NCP-5'linker (left), NCP-3'linker (center), and NCP-601 (right). The resolution range is shown in the box. Black arow indicates the interaction between UHRF1 TTD and acidic patch. **(B)** 3D classification of UHRF1 bound to the NCP-5'linker. The lower panels display the densities of the NCP and the UHRF1 TTD domain, with red indicating high density and yellow indicating low density. **(C)** Potential residues of the UHRF1 TTD domain that interact with the acidic patch of the NCP. Arg247, Arg250 and Arg 270 are represented as green stick models and highlighted with red dashed circles.

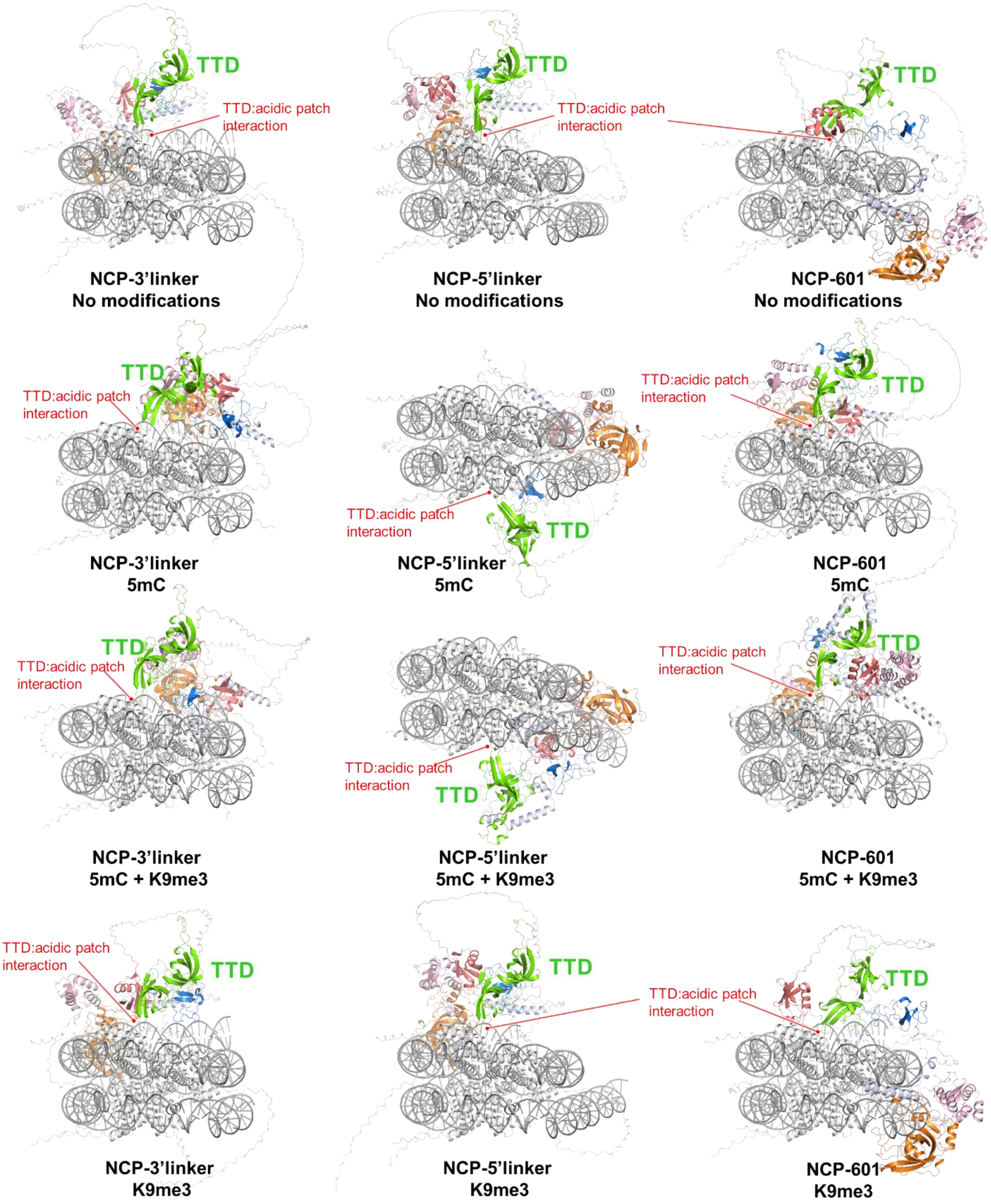

Supplementary Figure S9. AlphaFold3 structural predictions.

Predicted structures of UHRF1 in complex with NCP-5’linker, NCP-3’linker, and NCP-601 with or without 5mC in DNA and K9me3 in histone H3. NCPs are shown in gray. UHRF1 domains are color-coded as follows: UBL (salmon), TTD (green), PHD finger (light blue), SRA (orange), and RING (light pink). The predicted structures suggest that the TTD interacts with the acidic patch on NCP disk surface regardless of the presence of 5mC or H3K9me3 modifications.

Supplementary Table 1.

DNA sequence used for nucleosome core particle reconstruction

Name	Sequence (sense strand)	Primer	Oligo nucleotide
NCP-5'linker	ATCTGGGCC**M**GCCATATCAGAATC<br>CCGGTGCCGAGGCCGCTCAATTG<br>GTCGTAGACAGCTCTAGCACCGC<br>TTAAACGCACGTACGCGCTGTCC<br>CCCGCGTTTTAACCGCCAAGGGG<br>ATTACTCCCTAGTCTCCAGGCACG<br>TGTCAGATATATACATCGAT<br>(160 bp)	Forward: 5'-ATCTGGG<br>CC**M**GCCATATCAGAA<br>TCCCGGTGCCGAGG<br>CCG<br><br>Reverse: 5'-ATCGATG<br>TATATATCTGACACGT<br>GC	Not applicable
NCP-3'linker	ATCAGAATCCCGGTGCCGAGGCC<br>GCTCAATTGGTCGTAGACAGCTCT<br>AGCACCGCTTAAACGCACGTACG<br>CGCTGTCCCCCGCGTTTTAACCG<br>CCAAGGGGATTACTCCCTAGTCTC<br>CAGGCACGTGTCAGATATATACAT<br>CGATCC**M**GCAGGCC<br>(157bp)	Forward: 5'- ATCAGAA<br>TCCCGGTGCCGAGG<br>CCGC<br><br>Reverse: 5'- ATCCGTC<br>TCCATCGATGTATATA<br>TC	Forward: 5'-CGATC<br>C**M**GCAGGGCAG<br><br>Reverse: 5'-CTGCC<br>CTGCGGG
NCP-601	ATCAGAATCCCGGTGCCGAGGCC<br>GCTCAATTGGTCGTAGACAGCTCT<br>AGCACCGCTTAAACGCACGTACG<br>CGCTGTCCCCCGCGTTTTAACCG<br>CCAAGGGGATTACTCCCTAGTCTC<br>CAGGCACGTGTCAGATATA**M**GCAT<br>CGATGCAGG<br>(150 bp)	Forward: 5'- ATCAGAA<br>TCCCGGTGCCGAGG<br>CCGC<br><br>Reverse: 5'-TCTCAGA<br>TATCCCGTCTCGCGT<br>ATATCTGACACGTGC<br>CTG	Forward: 5'- TA**M**GC<br>ATCGATGCAGG<br><br>Reverse: 5'-CCTGC<br>ATCGATG

*M:5-methylcytosine. Underline shows the Widom 601 sequence
